# Policy Frameworks and Governance of Student Evaluation of Teaching in Nursing Education: Document Analysis

**DOI:** 10.1002/nop2.70863

**Published:** 2026-09-29

**Authors:** Mneera Alshammari, Kaye Rolls, Steven Frost, Peta Drury

**Affiliations:** ^1^ School of Nursing, Faculty of Science, Medicine & Health University of Wollongong New South Wales Australia

**Keywords:** document analysis, higher education, nursing academics, nursing education, policy, student evaluation of teaching

## Abstract

**Aim:**

To examine institutional documents on Student Evaluation of Teaching implementation in nursing schools and explore how policy frameworks influence their governance and use:

**Design:**

A qualitative document analysis.

**Methods:**

Institutional documents related to student evaluation of teaching implementation, quality assurance and programme evaluation were purposively selected from two nursing schools. Documents produced between 2023 and 2024 and relevant to student evaluation of teaching processes in nursing education were included. Data were systematically extracted using a structured analytical framework and thematically analysed.

**Data Sources:**

Six institutional policy and quality documents from two nursing schools were analysed.

**Results:**

Five themes were identified: enhancing educational quality, fostering continuous improvement, promoting accountability, adopting inclusive evaluation approaches, and aligning with accreditation‐driven strategic planning. Policies positioned student evaluation of teaching primarily as instruments for accountability and accreditation, often prioritising compliance over direct teaching enhancement.

**Conclusion:**

SETs function as policy tools supporting quality assurance and institutional performance. However, their current use may limit their potential to enhance teaching practice and faculty development. Strengthening feedback mechanisms and promoting faculty engagement in evaluation processes may enable SETs to contribute more meaningfully to educational improvement.

**Implications for Nursing Practice and Education:**

Integrating student evaluation findings into faculty development and curriculum design can strengthen teaching quality and support student‐centred learning in nursing education. These improvements contribute to preparing a competent nursing workforce capable of addressing complex healthcare needs. The findings highlight the need for balanced policy approaches that support both accountability and pedagogical development, with relevance for nursing education systems internationally.

**Impact:**

This study highlights how policy, governance and accreditation shape Student Evaluation of Teaching in nursing education. The findings may inform nursing schools, academics and policymakers in strengthening evaluations for both accountability and teaching improvement.

**Reporting Method:**

This study adhered to the Standards for Reporting Qualitative Research (SRQR).

**Patient or Public Contribution:**

No patient or public contribution was involved in this study.

## 1 Introduction

Student Evaluation of Teaching (SET) is widely recognised as a key mechanism for evaluating the quality of tertiary education (Stein et al. 2021). They provide a structured process for gathering student feedback on course content, teaching approaches and overall learning experiences (Joshi et al. 2024). When underpinned by clear policies and constructive use, SETs can drive continuous improvement by enabling educators and administrators to refine teaching practices, support faculty development, and strengthen academic programmes (Curby et al. 2020).

The effectiveness of SET, however, is closely tied to the robustness of policies guiding their implementation (Alsabahi 2020). Strong frameworks are required to ensure transparency, promote constructive feedback use and integrate evaluation results into professional development and curricular refinement (Ghofrani et al. 2023). In nursing education, this is especially important given the need to prepare graduates for both clinical competence and accreditation‐driven outcomes. Examining how policies shape the use of SETs can therefore provide insights of value not only in national contexts but also in global discussions on quality assurance, accreditation and faculty support in higher education (Aburizaizah 2022).

## 2 Background

Across diverse contexts, higher education systems are shifting from expansion toward prioritising educational quality. In Saudi Arabia, policy reforms such as Vision 2030 emphasise producing graduates with workforce‐relevant skills, positioning Student Evaluation of Teaching surveys (SETs) as a central component of institutional accountability and accreditation processes (Al Taweel 2020; Bataeineh and Aga 2023). Within this reform agenda, SETs are embedded in institutional quality assurance systems, particularly in nursing education, where they serve a dual role: evaluating teaching effectiveness and providing evidence for accreditation (Ruiz‐Mallén and Heras 2020), By systematically analysing SET results, institutions demonstrate compliance with standards set by the National Center for Academic Accreditation and Evaluation (NCAAA), supporting accountability and alignment with national and international benchmarks (Almurayh et al. 2022; Alqahtani et al. 2023).

This role parallels the Australian higher education context, where the Tertiary Education Quality and Standards Agency (TEQSA) oversees provider registration, accredits courses for non–self‐accrediting institutions, and compliance with the Higher Education Standards Framework (Baker 2025). Across both contexts, SETs function as key mechanisms for quality assurance, institutional monitoring and evidence‐based decision‐making.

In nursing education, these dynamics are significant due to the need to prepare graduates for safe clinical practice while meeting accreditation and regulatory standards. Policies that support the constructive use of SETs in faculty development, mentoring and curriculum design are therefore essential for enhancing teaching quality and ensuring alignment with professional expectations.

Despite the widespread use of SETs, limited research has examined how institutional policy frameworks govern their implementation in nursing education, particularly within non‐Western contexts such as Saudi Arabia. Existing studies have largely focused on student perceptions or psychometric properties of evaluation tools, with less attention given to how policies shape the purposes, processes and use of SETs within institutional quality assurance systems. Understanding these policy‐level dynamics is important for clarifying how evaluation systems support faculty development, accountability and educational improvement in nursing programmes.

### 2.1 Significance of the Study

These findings contribute to a deeper understanding of how evaluation systems can support both accountability and pedagogical development. The study provides implications for nursing education policy, faculty evaluation practices and quality assurance processes, informing how SETs may be used to support teaching improvement, accreditation requirements and professional preparation in nursing programmes. These findings can therefore provide insights relevant to similar higher education and nursing education contexts where SETs are used within quality assurance frameworks.

### 2.2 Research Aim

This study aimed to explore how institutional policy frameworks shape the implementation, governance and use of Student Evaluation of Teaching (SET) surveys in nursing education, using Saudi universities as a case example.

### 2.3 Research Objectives

The study aims to:

1. Identify the objectives articulated in policy documents regarding the purposes of student evaluations.
2. Examine how student evaluations are organised and administered within institutional frameworks
3. Explore how evaluation results are reported and utilised to inform quality assurance, accreditation and teaching improvement.

## 3 Method

### 3.1 Design

Document review is a qualitative method used to analyse institutional texts for patterns, themes and underlying assumptions (Bowen 2009; Morgan 2022). By systematically reviewing official reports, policy documents and institutional guidelines, it identifies themes relevant to the research focus and can be applied alone or alongside other methods (Cardno 2018). In this study, document review was employed to explore how Student Evaluation of Teaching surveys (SETs) are operationalised in nursing education, examining contextual factors, institutional processes and governance practices. The study adhered to the Standards for Reporting Qualitative Research (SRQR) to ensure comprehensive and transparent reporting (O'Brien et al. 2014).

### 3.2 Framework

This study adopted the document analysis framework proposed by Moilanen et al. (2022), further refined for nursing education research (Alshammari et al. 2025; Moilanen et al. 2022). The framework provides a systematic and rigorous process for analysing institutional documents through sequential and iterative phases, enabling in‐depth examination of policy structures, processes and outcomes (Table 1).

**TABLE 1:** Analytical framework for document review adapted from Moilanen et al. (2022).

Phase	Method	Action
1	Identifying the purpose	The review began with a clear definition of its purpose: to understand the role of SETs in nursing education and identify the key impact areas.
2	Determining the selection strategy	A strategic approach was developed to select the most relevant documents to the research questions. This involved contacting nursing schools and obtaining documents that could provide meaningful insights into SETs.
3	Developing an extraction matrix	An Excel table was developed to systematically record and analyse the documents' content. Table 2 was tailored to capture data related to the identified themes.
4	Conducting pilot testing	Before the data extraction commenced, pilot testing was conducted to refine the extraction process and ensure that the Excel file effectively recorded the necessary information from the documents for thematic analysis.
5	Collecting and Analysing Data	The documents were analysed using thematic analysis, a qualitative method that involves identifying, analysing and reporting patterns (themes) within the data. This process began with a thorough reading of the documents to familiarise researchers with the content and identify initial ideas for coding.
6	Ensuring credibility	Two researchers independently reviewed the documents and recorded key information in separate tables. They then convened to reconcile and finalise the data into a single comprehensive table for analysis.
7	Addressing ethical considerations	Finally, ethical considerations were carefully addressed, particularly in handling sensitive information and ensuring that the analysis represented the content of the documents.

### 3.3 Planning and Preparation Phase

The research aim and objectives guided the scope of the document review. The study focused on examining institutional policies related to SET implementation in nursing education. Early familiarisation with the context of Saudi higher education and accreditation requirements informed the selection of relevant documents and ensured alignment with the research purpose.

### 3.4 Document Identification and Selection

The document review was conducted between April and June 2024 in two nursing schools within Saudi universities. Of the seven schools approached, two granted access to relevant materials. A purposive sampling strategy was employed to select documents that were directly relevant to SET implementation, quality assurance processes and programme evaluation.

Inclusion criteria comprised institutional policy manuals, quality assurance reports and programme‐related documents explicitly addressing SET processes (Cardno 2018). Documents not directly related to SET implementation or lacking relevance to nursing education were excluded. Access to documents was facilitated by quality directors within each institution, ensuring relevance and credibility of the selected materials.

The inclusion of six documents from two institutions reflects the limited availability of accessible governance materials rather than an attempt to achieve institutional representativeness. Because five invited schools declined participation, the sample may be subject to selection bias, with participating institutions potentially reflecting programmes that have more structured quality assurance processes or greater willingness to share internal documentation. These institutions may therefore represent more developed governance contexts rather than the broader national landscape. Accordingly, the findings should be interpreted as an exploratory examination of SET governance within participating schools. Despite the small sample, the selected documents were institutional‐level policy and quality assurance materials that define formal evaluation structures, decision‐making pathways and oversight mechanisms, providing a concentrated view of how SET governance is formally organised within these settings.

### 3.5 Contextual and Preliminary Review

All documents were initially reviewed to gain an overall understanding of their structure, purpose and context. This preliminary reading enabled familiarisation with institutional language, policy intent and governance processes. Notes were taken to identify key concepts and recurring patterns, which informed subsequent stages of analysis.

### 3.6 Coding and Thematic Analysis

Data from six documents were systematically extracted using a structured data extraction table to ensure consistency and transparency (Table 2). The data extracted from the documents were analysed thematically to identify patterns and meaning (Braun and Clarke 2006) as two researchers independently coded the data, identifying meaningful units related to SET implementation, governance and use. Codes were grouped into categories and refined into themes through iterative discussion. This process enabled the development of themes that captured how SETs were framed, implemented, and utilised within institutional contexts.

**TABLE 2:** Summarised extraction table of institutional documents.

Document (code)	No. of docs	Title/type	Source	Key elements
D1	2	Annual program report (Report)	Nursing school	Reported enrolment, graduation outcomes and KPIs. Identified teaching strengths and areas for improvement. Set plans for curriculum renewal and faculty development.
D2	2	Program specification (report)	Nursing school	Defined programme goals, competencies and outcomes. Outlined curriculum structure and clinical placements. Linked assessments to accreditation standards.
D3	2	Quality system manual (manual)	Nursing school	Focused on compliance with national/international standards. Required KPIs, surveys and audits for quality monitoring. Applied staged cycle of planning, implementation and review.

### 3.7 Critical Interpretation and Synthesis

Themes were interpreted in relation to the broader policy and institutional context to generate deeper insights into the role of SETs in nursing education. The analysis moved beyond description to examine how policies shape practices, highlighting tensions between accountability, accreditation and teaching improvement. Findings were synthesised to provide a coherent understanding of how SET frameworks operate within and across institutions.

### 3.8 Ensuring Trustworthiness and Rigour

Several strategies were employed to enhance the trustworthiness of the study. Credibility was strengthened through independent data extraction and coding conducted by two researchers, followed by regular comparison and discussion of coding decisions. Any discrepancies were resolved through consensus, ensuring that interpretations were grounded in the data. Dependability was maintained by using a structured data extraction table and applying a systematic and transparent analytical process across all documents. This ensured consistency in how data were identified, recorded and analysed. An audit trail was maintained throughout the study, documenting key stages of data extraction, coding, theme development and decision‐making processes, thereby enabling transparency and traceability of the analysis. Reflexivity was addressed by ongoing reflection on the researchers' familiarity with the educational context and potential influence on interpretation. Reflexive discussions were undertaken during analysis to minimise bias and support balanced interpretation of the findings.

### 3.9 Ethical Considerations

Ethical approval was not required, as the study involved institutional documents rather than human participants. Formal permission was obtained from participating institutions, and all documents were handled confidentially and used solely for research purposes.

## 4 Findings

### 4.1 Results of Document Analysis

Data extracted and organised in Table 2 were used to address the research questions. Discrepancies in interpretation were discussed and resolved by consensus before consolidating the findings into a single comprehensive table. Document statements were examined in relation to policy objectives, implementation processes and the use of results, capturing key aspects of Student Evaluation of Teaching (SET) governance. Information was organised by institutional approaches, including the evaluation tools employed, methods of survey administration, and the ways in which results were used to inform teaching and learning.

The findings were synthesised into a cyclical framework (Figure 1) that depicts SET policies as an iterative process in which objectives guide implementation, implementation generates outcomes and outcomes feed back into policy development and quality enhancement.

**FIGURE 1:**
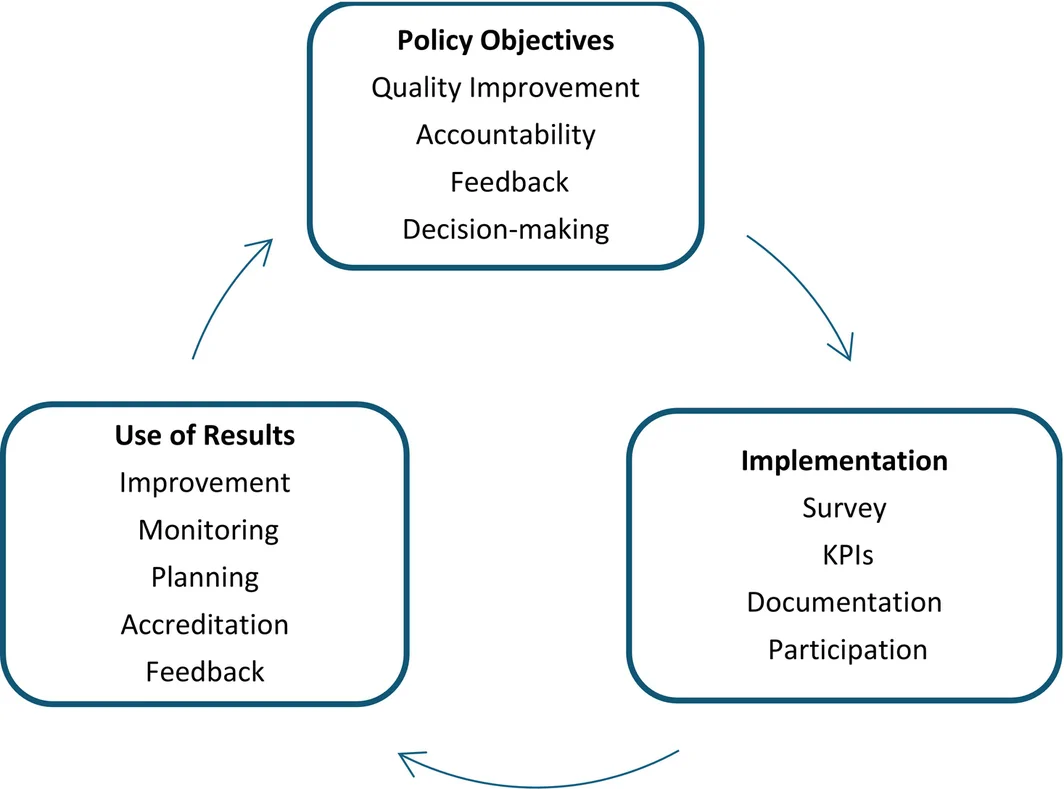
Cyclical framework of Student Evaluation of Teaching (SET) policies in nursing education, illustrating the iterative process through which policy objectives guide implementation, implementation generates outcomes and outcomes feed back into policy refinement and quality enhancement.

### 4.2 Thematic Analysis

The analysis began with familiarisation, during which each document was read and re‐read to develop a comprehensive understanding of its content (Nowell et al. 2017). Preliminary notes and highlights were made around sections relevant to the research questions, forming the foundation for the coding process.

A systematic coding procedure was then applied, classifying relevant data segments with concise descriptors that captured their meaning (Maguire and Delahunt 2017). Deductive coding, guided by the research questions (Dawson et al. 2024), ensured close alignment with the study's objectives. Codes were subsequently reviewed and grouped into broader categories that reflected institutional practices and policy priorities.

These categories informed the development of overarching themes. For example, codes relating to *educational quality, continuous improvement and learning outcomes* clustered under the theme Enhancement of Educational Quality. Similarly, codes such as *surveys, evaluation tools, documentation and participation* contributed to the theme Inclusive and Comprehensive Evaluation Methods. Codes linked to *quality improvement, performance monitoring, accreditation and stakeholder feedback* formed the basis of the themes Accountability and Decision‐Making and Strategic Planning and Accreditation. Through iterative comparison and refinement, themes were checked for coherence, overlap was addressed, and broader categories were divided where necessary (Williams and Moser 2019).

### 4.3 Results of Thematic Analysis

The analysis identified five overarching themes that demonstrate how policies shape the effective uptake of Student Evaluation of Teaching (SET) surveys.

#### 4.3.1 Theme 1: Enhancement of Educational Quality

Enhancing educational quality emerged as a primary focus of SET policies, with an emphasis on assessing learning outcomes and programme quality to uphold high standards. This ensures that educational practices positively impact student success. For example, as documented in D1, “*the program is successful…providing positive program quality*” and demonstrates a “*high level of satisfaction…delivering a quality program*” (D1). These findings indicate that policies driving the implementation of SETs reinforce quality standards as an institutional priority. Similarly, assessment methods outlined in D2 “*measure the achievement of program learning outcomes*” (D2), reflecting how SET data provides actionable insights for maintaining excellence in educational delivery.

#### 4.3.2 Theme 2: Continuous Improvement and Feedback

Policies supporting continuous improvement rely heavily on feedback mechanisms derived from SETs, which inform curriculum revisions and teaching strategies. SETs act as a cornerstone for identifying strengths and areas for enhancement. For instance, D1 states that evaluations “*reflect the program's commitments to delivering a high‐quality program*” while identifying “*continuous improvement…areas for improvement*” (D1). Furthermore, D3 highlights the importance of student input in policy‐driven feedback systems, noting “*satisfaction with adequacy and diversity of learning sources*” and students' “*overall rating for the quality of courses on a Five‐point scale in the annual survey*” (D3).

#### 4.3.3 Theme 3: Accountability and Decision‐Making

SET‐driven policies bolster accountability by facilitating informed decision‐making and supporting programme development. The structured use of SETs ensures transparency in teaching evaluations and fosters continuous refinement of teaching strategies. For instance, D3 highlights “*mechanisms for evaluating the effectiveness of field experience courses*” and the “*criteria for different assessment tools used in theory or field experience courses*” (D3), which validate the objectivity of assessment practices. Moreover, the process “*follows up program improvement plans*” (D3), underscoring how policies mandating SET reviews align institutional activities with strategic goals.

#### 4.3.4 Theme 4: Inclusive and Comprehensive Evaluation Methods

Inclusive and comprehensive SET policies utilise diverse tools and approaches to ensure a holistic assessment of educational quality. This theme highlights the importance of adopting policies that mandate multi‐faceted evaluation methods. For example, D2 mentions “*course evaluation surveys by students*” along with “*field experience evaluation surveys*” and “*program‐level indirect assessment methods*” (D2). Similarly, D3 underscores the integration of “*student experience surveys, program evaluation surveys and course evaluation surveys*,” which together provide a comprehensive understanding of programme effectiveness (D3).

#### 4.3.5 Theme 5: Strategic Planning and Accreditation

Strategic planning and accreditation are directly influenced by SET policies, which provide actionable insights to inform institutional development plans. Policies supporting the structured use of SETs ensure alignment with accreditation standards and institutional goals. For instance, D3 mentions actions such as “*taking corrective actions in the light of the DQD [deanship of quality and development] review report*” and “*preparing reports for results of the evaluation of internal quality systems for programs*” (D3). Additionally, discussions in committees to “*follow up program improvement plans*” and “*incorporate the findings into the program improvement plan*” (D3) illustrate how SET data is operationalised through policies to drive accreditation efforts.

## 5 Discussion

### 5.1 SETs in Nursing Education

The findings from this document review highlight the multifaceted role of Student Evaluation of Teaching (SET) surveys in higher education, with nursing schools providing a clear example of their dual function as both feedback mechanisms and instruments of institutional accountability. Internationally, SETs are commonly used to support educational quality and align teaching practices, curriculum design and learning environments with internal benchmarks and external accreditation requirements (Aljendan 2024; Nazir et al. 2020). By embedding structured feedback systems within policy frameworks, universities may support standards, respond to accreditation demands and promote continuous improvement in response to changing educational contexts (Alzahrani et al. 2021).

ETs also facilitate systematic evaluation of teaching effectiveness, course design and programme quality by drawing on student experiences (Aljendan 2024). Such initiatives may support the adoption of student‐centred pedagogies, encouraging adaptability, innovation and alignment with learner preferences for more active and engaging approaches to education (Joshi et al. 2024). Beyond individual teaching practices, SETs also play a pivotal role in sustaining curriculum relevance across higher education. By identifying areas where content requires updating, institutions can align curricula with industry trends and labour market demands (Al Taweel 2020). Such revisions may strengthen graduate preparedness for professional environments and ensure that nursing and other health programmes remain competitive and responsive to societal needs (Ulker 2021).

### 5.2 Student Engagement in Decision‐Making

Student involvement in evaluation processes can promote a student‐centred approach to quality assurance by integrating learner perspectives into institutional decision‐making (Aljendan 2024). This collaboration may foster trust and engagement, creating environments where students feel valued and invested in their education. Recent research highlights that meaningful student voice mechanisms strengthen partnerships between students and institutions, with trust‐building and inclusive dialogue identified as essential for translating student feedback into institutional change (McConnell et al. 2025). Such approaches can enhance transparency and promote shared responsibility for educational improvement.

Student engagement has also been increasingly recognised as a multidimensional construct encompassing relational, participatory and proactive involvement in academic communities, all of which influence institutional decision‐making and educational quality (Veiga et al. 2026). When students actively contribute to evaluation systems, institutions may gain richer insights into learning experiences and can align policies with student needs and expectations. Within health and professional education, including nursing, student engagement is gaining momentum as a strategy that can improve programme performance and strengthen policy development (Neshastesaz Kashi et al. 2024). Evidence suggests that incorporating student perspectives into governance and quality assurance processes can support more responsive and contextually relevant educational decisions (Neshastesaz Kashi et al. 2024). At the same time, SETs can also contribute to institutional accountability, with universities using feedback to address areas requiring attention and demonstrate their commitment to excellence for students, faculty and accrediting bodies (Aburizaizah 2022).

### 5.3 Limitations of Current Policy Approaches

Despite these benefits, the reviewed documents suggested a stronger emphasis on compliance and procedural rigour. Program evaluation reports and quality assurance manuals provide extensive technical detail but provided limited explicit guidance tailored to the everyday realities of faculty work. This emphasis may limit adaptability to the evolving needs of educators and students (Ulker 2021). Evaluation mechanisms such as surveys and Key Performance Indicators (KPIs), while comprehensive, may not fully capture the qualitative dimensions of teaching effectiveness or the relational nuances of faculty‐student interaction (Laundon et al. 2023). Instruments like the Course Evaluation Survey (CES) and Program Evaluation Survey (PES) yield valuable data, yet their utility may be limited when findings are not clearly translated into practical support for teaching and learning improvement.

Nevertheless, the analysed documents provided limited explicit attention to integrating evaluation results into collaborative, faculty‐led discussions or workshops. This may reduce opportunities for meaningful dialogue and shared ownership of the improvement process among educators (Cook et al. 2024). A commonly noted concern in higher education is the reliance on quantitative metrics and standardised processes, which risks overlooking the diversity of academic practices and the discipline‐specific needs of faculties such as nursing (Laundon et al. 2023). While surveys generate useful insights into student satisfaction and programme quality, they may not fully capture the complexity of teaching and learning dynamics (Hutchinson et al. 2024) Similarly, detailed documentation, such as quality files and performance indicators, enhances transparency and accountability but may become bureaucratic processes that place additional demands on faculty rather than empower them to improve practice (Daskalopoulou 2024).

### 5.4 Study Implications

A key strength of this study lies in its focus on the underexplored area of policy frameworks guiding Student Evaluation of Teaching (SET) within nursing education, particularly outside Western contexts. By systematically analysing institutional documents using a structured analytical framework, the study provides a rigorous approach that may be applied in similar higher education settings to examine policy environments. The use of thematic synthesis enabled the identification of nuanced insights into how SETs are positioned within governance and quality assurance processes. These findings offer preliminary, context‐specific insights into SET governance within participating institutions and may inform future research exploring evaluation frameworks in comparable educational contexts.

### 5.5 Limitations

Nevertheless, the analysis was restricted to documents from two nursing schools, which may not capture the broader range of policies and practices shaping SET implementation nationally or internationally. In addition, restricted access to certain institutional documents may have resulted in the omission of relevant policies or guidelines, thereby narrowing the scope of analysis. The inclusion of six documents from two institutions reflects the availability of accessible governance materials rather than an attempt to achieve institutional representativeness. Because participation depended on institutional willingness to provide internal quality assurance documentation, the included schools may reflect programmes with more established evaluation processes or greater transparency in governance structures. This introduces the possibility of selection bias and limits the generalizability of the findings. However, the analysed documents were governance‐level materials, including quality manuals, programme specifications and annual reports, which outlined formal structures, decision‐making pathways and implementation mechanisms for SET use. These document types provide insight into how evaluation processes are embedded within institutional quality systems. Accordingly, the dataset offers a focused snapshot of governance frameworks within participating institutions, allowing exploration of policy intent and structural approaches while acknowledging that practices may vary across other settings.

### 5.6 Recommendations for Further Research

Future research should include a broader range of institutions to develop a more comprehensive understanding of how SET are governed and operationalised across different contexts. Comparative studies may help identify similarities and differences in policy frameworks and highlight context‐specific challenges. Further work is also needed to examine how SET policies are enacted in practice, for example through interviews or focus groups with faculty, administrators and students, to explore how governance frameworks influence day‐to‐day teaching and learning. Additionally, research could investigate how evaluation outcomes are integrated into faculty development and curriculum improvement initiatives. Such studies would provide deeper insight into how SET policies are implemented and refined within nursing education and comparable higher education settings.

## 6 Conclusion

This qualitative document review explored how Student Evaluation of Teaching (SET) is positioned within governance frameworks in nursing education, highlighting their role in quality assurance, institutional improvement and accreditation processes. The findings suggest that SET is embedded within institutional evaluation systems, while faculty engagement and pedagogical development were less explicitly articulated in the analysed documents. These observations indicate opportunities to strengthen policy frameworks by more clearly linking SET findings to professional development, collaborative teaching improvement and balanced accountability mechanisms. As an exploratory analysis of governance documents from participating institutions, the study provides contextual insight into how SETs are formally structured within nursing education and offers a foundation for future research examining policy implementation and development across similar higher education contexts.

## Author Contributions

**Mneera Alshammari:** conceptualization, investigation, writing – original draft, writing – review and editing, visualization, methodology, formal analysis, data curation, resources. **Kaye Rolls:** conceptualization, methodology, supervision, formal analysis, investigation. **Steven Frost:** conceptualization, methodology, supervision. **Peta Drury:** conceptualization, writing – review and editing, methodology, supervision, formal analysis.

## Funding

The authors have nothing to report.

## Disclosure

The authors declare that no artificial intelligence‐assisted technologies were used in the preparation of this manuscript.

## Ethics Statement

Ethical approval was not required for this study, as confirmed through consultation with the participating universities. Institutional permission was obtained to access documents, and all procedures adhered to institutional protocols for document use and data management.

## Conflicts of Interest

The authors declare no conflicts of interest.

## Data Availability

The data that support the findings of this study are available on request from the corresponding author. The data are not publicly available due to privacy or ethical restrictions.

## References

[nop270863-bib-0001] Aburizaizah, S. J. 2022. “The Role of Quality Assurance in Saudi Higher Education Institutions.” International Journal of Educational Research Open 3: 100127.

[nop270863-bib-0002] Al Taweel, I. R. 2020. “The Impact of Higher Education Outcomes on Labor Market Requirements Under the Vision of Saudi Arabia 2030.” International Journal of Advanced and Applied Sciences 7, no. 6: 110–115.

[nop270863-bib-0003] Aljendan, A. Y. 2024. “Improving Teaching Quality in Higher Education Through Student Evaluation Feedback in Saudi Universities.” Educational Administration: Theory and Practice 30, no. 2: 83–100.

[nop270863-bib-0004] Almurayh, A. , S. Saeed , N. Aldhafferi , A. Alqahtani , and M. Saqib . 2022. “Sustainable Education Quality Improvement Using Academic Accreditation: Findings From a University in Saudi Arabia.” Sustainability 14, no. 24: 16968.

[nop270863-bib-0005] Alqahtani, A. , A. Makki , and R. Abdulaal . 2023. “A Proposed NCAAA‐Based Approach to the Self‐Evaluation of Higher Education Programs for Academic Accreditation: A Comparative Study Using TOPSIS.” Decision Science Letters 12, no. 2: 333–352.

[nop270863-bib-0006] Alsabahi, R. 2020. “Problematizing Student Evaluation of Teaching in Saudi Arabia: Merits, Demerits and Impacts on Performance.” Critical Issues in Teaching English and Language Education: International Research Perspectives: 373–400.

[nop270863-bib-0007] Alshammari, M. , K. Rolls , S. A. Frost , and P. Drury . 2025. “Using Document Review for Pedagogical Research: A Practical Guide for Nursing Educators.” Evidence‐Based Nursing 29. 10.1136/ebnurs-2025-104337 40835406

[nop270863-bib-0008] Alzahrani, B. , H. Bahaitham , M. Andejany , and A. Elshennawy . 2021. “How Ready Is Higher Education for Quality 4.0 Transformation According to the LNS Research Framework?” Sustainability 13, no. 9: 5169.

[nop270863-bib-0009] Baker, A. T. 2025. “Quality Assurance in Australian Higher Education.” In Navigating Quality Assurance and Accreditation in Global Higher Education, 457–478. IGI Global Scientific Publishing.

[nop270863-bib-0010] Bataeineh, M. , and O. Aga . 2023. “Integrating Sustainability Into Higher Education Curricula: Saudi Vision 2030.” Emerald Open Research 1, no. 3.

[nop270863-bib-0011] Bowen, G. A. 2009. “Document Analysis as a Qualitative Research Method.” Qualitative Research Journal 9, no. 2: 27–40.

[nop270863-bib-0012] Braun, V. , and V. Clarke . 2006. “Using Thematic Analysis in Psychology.” Qualitative Research in Psychology 3, no. 2: 77–101.

[nop270863-bib-0013] Cardno, C. 2018. “Policy Document Analysis: A Practical Educational Leadership Tool and a Qualitative Research Method.” Educational Administration: Theory & Practice 24, no. 4: 623–640.

[nop270863-bib-0014] Cook, S. , D. Watson , and R. Webb . 2024. “Performance Evaluation in Teaching: Dissecting Student Evaluations in Higher Education.” Studies in Educational Evaluation 81: 101342.

[nop270863-bib-0015] Curby, T. , P. McKnight , L. Alexander , and S. Erchov . 2020. “Sources of Variance in End‐Of‐Course Student Evaluations.” Assessment and Evaluation in Higher Education 45, no. 1: 44–53.

[nop270863-bib-0016] Daskalopoulou, A. 2024. “Understanding the Impact of Biased Student Evaluations: An Intersectional Analysis of Academics' Experiences in the UK Higher Education Context.” Studies in Higher Education 49, no. 12: 2411–2422.

[nop270863-bib-0017] Dawson, S. , J. Goulding , and B. Gledson . 2024. “Use of Focus Groups and Reflexive Thematic Analysis to Uncover the Essential Conditions for UK Built Environment Degree Apprenticeships.” International Journal of Construction Education and Research 20, no. 4: 562–580. 10.1080/15578771.2024.2404966.

[nop270863-bib-0018] Ghofrani, M. , L. Valizadeh , V. Zamanzadeh , A. Ghahramanian , A. Janati , and F. Taleghani . 2023. “Baccalaureate Nursing Education Institutions' Key Performance Indicators: A Review of the Existing Indicators and Qualitative Analysis of Expert Interviews.” BMC Nursing 22, no. 1: 357. 10.1186/s12912-023-01484-6 PMC10552274 37798710

[nop270863-bib-0019] Hutchinson, M. , R. Coutts , D. Massey , et al. 2024. “Student Evaluation of Teaching: Reactions of Australian Academics to Anonymous Non‐Constructive Student Commentary.” Assessment and Evaluation in Higher Education 49, no. 2: 154–164.

[nop270863-bib-0020] Joshi, P. R. , P. R. Pant , and R. B. Chand . 2024. “Student Evaluation of Teaching: A Tool for the Assessment of Teaching Learning Practices in Higher Education Institutions.” KMC Journal 6, no. 1: 192–209.

[nop270863-bib-0021] Laundon, M. , S. Cunningham , and A. Cathcart . 2023. “Institutional Approaches to Evaluation of Learning and Teaching: A Sector Scan of Australasian Universities.” Journal of Higher Education Policy and Management 45, no. 5: 511–528.

[nop270863-bib-0022] Maguire, M. , and B. Delahunt . 2017. “Doing a Thematic Analysis: A Practical, Step‐By‐Step Guide for Learning and Teaching Scholars.” All Ireland Journal of Higher Education 9, no. 3: 3351.

[nop270863-bib-0023] McConnell, C. , S. Smith , K. Olomu , E. King , O. Ghosh , and C. Hamshire . 2025. “‘My Input Was Actually Being Listened to and Could Lead to Real Change’: Developing Trust Through Student Voice in Student‐Staff Partnerships.” Student Engagement in Higher Education Journal 7, no. 1: 219–236.

[nop270863-bib-0024] Moilanen, T. , M. Sivonen , K. Hipp , et al. 2022. “Developing a Feasible and Credible Method for Analyzing Healthcare Documents as Written Data.” Global Qualitative Nursing Research 9: 23333936221108706. 10.1177/23333936221108706 PMC9272191 35832602

[nop270863-bib-0025] Morgan, H. 2022. “Conducting a Qualitative Document Analysis.” Qualitative Report 27, no. 1: 64–77.

[nop270863-bib-0026] Nazir, M. , A. Al‐Ansari , K. AlKhalifa , B. Gaffar , and J. AlHumaid . 2020. “Use of Student Evaluation of Teaching (SET) Survey to Evaluate Effectiveness of Teaching in a Leadership Course Among Dental Students Over Three Years.” Scientific World Journal 2020, no. 1: 6436102. 10.1155/2020/6436102 PMC7285415 32565752

[nop270863-bib-0027] Neshastesaz Kashi, H. , S. Goharinezhad , S. Soleimanpour , and A. H. Mohammadi . 2024. “Mapping Student Engagement in Health Professions Education Policy and Decision‐Making: A Scoping Review.” BMC Medical Education 24, no. 1: 325. 10.1186/s12909-024-05283-8. PMC10960467 38519931

[nop270863-bib-0028] Nowell, L. S. , J. M. Norris , D. E. White , and N. J. Moules . 2017. “Thematic Analysis: Striving to Meet the Trustworthiness Criteria.” International Journal of Qualitative Methods 16, no. 1: 1609406917733847.

[nop270863-bib-0029] O'Brien, B. C. , I. B. Harris , T. J. Beckman , D. A. Reed , and D. A. Cook . 2014. “Standards for Reporting Qualitative Research: A Synthesis of Recommendations.” Academic Medicine 89, no. 9: 1245–1251. 10.1097/ACM.0000000000000388 24979285

[nop270863-bib-0030] Ruiz‐Mallén, I. , and M. Heras . 2020. “What Sustainability? Higher Education Institutions' Pathways to Reach the Agenda 2030 Goals.” Sustainability 12, no. 4: 1290.

[nop270863-bib-0031] Stein, S. J. , A. Goodchild , A. Moskal , S. Terry , and J. McDonald . 2021. “Student Perceptions of Student Evaluations: Enabling Student Voice and Meaningful Engagement.” Assessment and Evaluation in Higher Education 46, no. 6: 837–851.

[nop270863-bib-0032] Ulker, N. 2021. “How Can Student Evaluations Lead to Improvement of Teaching Quality? A Cross‐National Analysis.” Research in Post‐Compulsory Education 26, no. 1: 19–37.

[nop270863-bib-0033] Veiga, F. , Z. Y. Wong , F. M. Veiga , et al. 2026. “Higher Education Student Engagement in the Academic Community: Clarifying the Concept and Proposing a Short Measure [Original Research].” Frontiers in Education 11–2026: 1773320. 10.3389/feduc.2026.1773320.

[nop270863-bib-0034] Williams, M. , and T. Moser . 2019. “The Art of Coding and Thematic Exploration in Qualitative Research.” International Management Review 15, no. 1: 45–55.

